# An Adaptive Prescribed Performance Tracking Motion Control Methodology for Robotic Manipulators with Global Finite-Time Stability

**DOI:** 10.3390/s22207834

**Published:** 2022-10-15

**Authors:** Anh Tuan Vo, Thanh Nguyen Truong, Hee-Jun Kang

**Affiliations:** Department of Electrical, Electronic and Computer Engineering, University of Ulsan, Ulsan 44610, Korea

**Keywords:** Uniform Second-Order Sliding Mode Observer, Prescribed Performance Control, robot manipulators, finite-time Stability

## Abstract

In this paper, the problem of an APPTMC for manipulators is investigated. During the robot’s operation, the error states should be kept within an outlined range to ensure a steady-state and dynamic attitude. Firstly, we propose the modified PPFs. Afterward, a series of transformed errors is used to convert “constrained” systems into equivalent “unconstrained” ones, to facilitate control design. The modified PPFs ensure position tracking errors are managed in a pre-designed performance domain. Especially, the SSE boundaries will be symmetrical to zero, so when the transformed error is zero, the tracking error will be as well. Secondly, a modified NISMS based on the transformed errors allows for determining the highest acceptable range of the tracking errors in the steady-state, finite-time convergence index, and singularity elimination. Thirdly, a fixed-time USOSMO is proposed to directly estimate the lumped uncertainty. Fourthly, an ASTwCL is applied to deal with observer output errors and chattering. Finally, an observer-based-control solution is synthesized from the above techniques to achieve PCP in the sense of finite-time Lyapunov stability. In addition, the precision, robustness, as well as harmful chattering reduction of the proposed APPTMC are improved significantly. The Lyapunov theory is used to analyze the stability of closed-loop systems. Throughout simulations, the proposed PPTMC has been shown to perform well and be effective.

## 1. Introduction

Increasing performance requirements are put into practice with a wide range of the robot’s applications [1] such as fire prevention, medical support, industrial assembly, etc. However, some general problems of mechanical systems the dynamical uncertainties such as state constraints, frictions, high nonlinearity, parametric variations, etc., are unavoidable in reality [2]. They can be also exterior disturbances leading to the robot system may perform poorly in transient and steady-state states, causing instability in the robot’s operation. Moreover, system uncertainties have highly complicated dynamics since their dynamics are influenced by the state of the system, its derivatives, and its inputs. Thus, it remains an open problem to determine an effective compensation method for system uncertainties in robot manipulators’ trajectory tracking control. Under the influence of time-varying disturbances, the traditional PID controllers [3,4] have difficulty in maintaining accurate tracking. Therefore, a few more advanced controllers such as the modified PID control [5,6], Sliding Mode Control (SMC) [7,8,9], Computed Torque Control (CTC) [10], Back-stepping Control Method (BsCM) [11], Adaptive Control Method (ACM) [12], and so on, have been widely used in control design to reduce the effects of system uncertainty. SMC is most used by the control community due to its robustness, accuracy, and ease of implementation. However, unknown terms must be suppressed by the SMC’s switching terms to ensure the existence of the sliding surface-reaching motion, leading to large chattering [13]. Moreover, it is unfortunate that most of these methods, including SMC, can only asymptotically converge to the neighborhood equilibrium points.

To obtain effective anti-disturbance ability and high tracking accuracy for robot systems with complicated dynamics and external disturbances, there are a lot of disturbance rejection control methods in the literature such as Sliding Mode Observer-based Control Method (SMO-CM) [14,15,16,17,18], Time-Delay Estimation-based Control Method (TDE-CM) [9,19], Disturbance Observer-based Control Method (DO-CM) [20], Active Disturbance Rejection Control Method (ADRCM) [21], and so on. In addition to removing the unreasonable assumption as H2 norm-bounded assumption [22], the SMO-CMs possess the robust control performance of the SMC methods. Using the SMC in conjunction with an observer, its switching part with a small sliding gain can compensate for the estimation error of Disturbance Observer (DO) along with minimizing chattering. This has prompted SMO-CM studies to become increasingly popular. Despite the fact that the SMO-CMs can offer powerful performance for controlled uncertain systems, most SMO-CMs employ asymptotical stability theory for their design. Therefore, those schemes only achieve asymptotical convergence. In control systems, fast/finite-time/fixed-time convergence is an important performance property. Finite-time/fixed-time convergence differs from asymptotic convergence in that the system states converge to zero in a finite amount of time or in fixed time. Therefore, the Finite-Time Control Method (FnTCM) [23,24] or the Fixed-Time Control Method (FxTCM) [17,25] could be achieved a better convergence rate and tracking precision.

Recently, a series of SMC with finite-time/fixed-time convergence have been introduced along with the expansion of FnTCM and FxTCM theory, such as Integral SMC (ISMC) [26,27], Terminal SMC (TSMC) [28,29], Non-singular TSMC (NTSMC) [30,31], Fast TSMC (FTSMC) [29,32,33], Fast NTSMC (FNTSMC) [34,35], and so on. Therefore, the Finite-Time Disturbance Observers (FnTDOs) or Fixed-Time Disturbance Observers (FxTDOs) have been developed such as Second-Order Sliding Mode Observer (SOSMO) [16,36], Uniform SOSMO (USOSMO) [37,38], or Third-Order Sliding Mode Observer (TOSMO) [14,39,40]. It can be seen from a comparison between FnTDO and FxTDO that under the same observer’s gains, FnTDO cannot achieve a similar fast convergence performance as FxTDO. With the FxTDO, system states and estimation errors have uniform convergence time, and their upper bounds are not affected by the system’s initial condition. The FxTDO is therefore a good candidate for handling unknown components. In addition, a combination of the FxTDO and the SMC also can minimize the effects of the chattering, as mentioned above.

In stabilization and tracking problems, transient performance is an important index for controlled systems that we need to concentrate on it. Though all of the conventional control methodologies can manipulate the state error variables to a residual set with an unknown size, it is not guaranteed convergence of trajectory states within a small maximum overshoot and maintained the steady states in a predefined boundary because of the lack of suitable techniques. The concept of the Prescribed Performance Control (PPC) was first proposed in [41] for satisfying transient behavior. That means both transient performance and steady-state performance are guaranteed with the following conditions: (1) tracking errors are limited to a small residual set; (2) the convergence rate is not less than a predetermined constant; (3) the maximum overshoot is limited to a predetermined space. Most current PPC studies [41,42,43] used a single Prescribed Performance Function (PPF) to generate boundaries of specific performance. For example, ref. [41] used a PPF P(t) to determine the operating space in which P(t) is prescribed as the upper boundary and −NP(t)(0<N<1) is prescribed as the lower boundary. This method has some drawbacks, as follows: the operating domain of specified performance be scaled down over a specified static error value because the lower boundary will be *N* times smaller than the upper boundary. In the steady state, these two boundaries will not be symmetrical about each other through zero if a ratio of PPF is used to create the lower boundary. Therefore, the transformed error can be converged to zero but the tracking error differs from zero. This situation presents a real challenge in choosing an Error Transformation Function (ETF). In addition, some ETFs [44,45,46] have a singularity problem, which negatively affects the operation of the real system.

Inspired by the mentioned investigation, we propose an Adaptive Prescribed Performance Tracking Motion Control (APPTMC) for robotic manipulators with global finite-time stability. Our achievements include:

- the proposed PPFs ensure position tracking errors are managed in a pre-designed performance domain. Especially, the Steady-State Error (SSE) boundaries will be symmetrical to zero, so when the transformed error is zero, the tracking error will be as well;
- a fixed-time USOSMO is proposed to directly estimate the lumped uncertainty;
- in addition to determining the highest acceptable range of tracking errors at the steady state, the modified Non-singular Integral Sliding Mode Surface (NISMS) can also eliminate singularities and achieve finite-time convergence;
- the Adaptive Super-twisting Control Law (ASTwCL) is applied to deal with observer output errors and chattering. In this way, the control design clears the upper boundary requirement of all uncertainty.
- the proposed APPTMC ensures the effective reduction of harmful chattering behaviors by active compensations;
- guarantees prescribed performance in the sense of finite-time Lyapunov stability;
- the effectiveness of the APPTMC has been fully confirmed through simulations.

Following is a summary of the rest of the article. Section 2 describes the related preliminaries and mathematical formulas for robot dynamics. Throughout Section 3, the USOSMO design and the APPTMC design are presented along with their combination to solve the tracking control problems. A discussion of innovative features is presented in Section 4 through simulation examples on a 3-Degrees of Freedom (DOF) robot manipulator. As a result of this research, we draw some important conclusions and look ahead to future research directions in Section 5.

A list of nomenclature is provided in Table 1 for the reader’s convenience. In addition, some other physical symbols will be fully defined in the paper.

## 2. Problem Statement

### 2.1. Dynamic Modeling of Robotic Manipulators

Dynamic modeling of an n-DOF robot manipulator is described as [2]:

(1) $$\begin{array}{c} H\left(p\right)\ddot{p}+C\left(p,\dot{p}\right)\dot{p}+g\left(p\right)+F_{r}\left(\dot{p}\right)=\tau -\tau_{d}, \end{array}$$

where $H\left(p\right)=H_{0}\left(p\right)+\delta H\left(p\right)\in R^{n\times n}$ is an inertial matrix that is nonsingular. $C\left(p,\dot{p}\right)=C_{0}\left(p,\dot{p}\right)+\delta C\left(p,\dot{p}\right)\in R^{n\times n}$ represent Centripetal and Coriolis matrix and $g\left(p\right)=g_{0}\left(p\right)+\delta g\left(p\right)\in R^{n\times 1}$ is gravity vector. $H_{0}\left(p\right)\in R^{n\times n}$, $C_{0}\left(p,\dot{p}\right)\in R^{n\times n}$, and $g_{0}\left(p\right)\in R^{n\times 1}$ symbolize the computed dynamic function of $H\left(p\right)$, $C\left(p,\dot{p}\right)$, and $g\left(p\right)$, respectively. $\delta H\left(p\right)\in R^{n\times n}$, $\delta C\left(p,\dot{p}\right)\in R^{n\times n}$, and $\delta g\left(p\right)\in R^{n\times 1}$ symbolize undefined dynamic function of $H\left(p\right)$, $C\left(p,\dot{p}\right)$, and $g\left(p\right)$, respectively. Friction forces, external disturbances, and control torques are represented by the vectors $F_{r}\left(\dot{p}\right)\in R^{n\times 1}$, $\tau_{d}\in R^{n\times 1}$, and $\tau \in R^{n\times 1}$, respectively.

Let $z={\left[z_{1},z_{2}\right]}^{T}={\left[p,\dot{p}\right]}^{T}$ and u=τ. then, the robot dynamics (1) can be described in form of the second-order state-space formula:

(2) $$\begin{array}{c} \left\{\begin{array}{c} {\dot{z}}_{1}=z_{2}\\ {\dot{z}}_{2}=J\left(z\right)u+W\left(z\right)-\Delta \left(z,\delta ,\tau_{d}\right) \end{array}\right., \end{array}$$

where $J\left(z\right)=H_{0}^{-1}\left(p\right)$, $W\left(z\right)=-H_{0}^{-1}\left(p\right)\left(C_{0}\left(p,\dot{p}\right)\dot{p}+g_{0}\left(p\right)\right)$ stands for the calculable or measurable terms and $\Delta \left(z,\delta ,\tau_{d}\right)=H_{0}^{-1}\left(p\right)\left(F_{r}\left(\dot{p}\right)+\delta H\left(p\right)\ddot{p}+\delta C\left(p,\dot{p}\right)\dot{p}+\delta g\left(p\right)+\tau_{d}\right)$ stands for the lumped unknown terms.

Let $z_{e}={\left[z_{e_{1}}^{T},z_{e_{2}}^{T}\right]}^{T}={\left[{\left[z_{1}-z_{d}\right]}^{T},{\left[z_{2}-{\dot{z}}_{d}\right]}^{T}\right]}^{T}$. So, Equation (2) is rewritten as:

(3) $$\begin{array}{c} \left\{\begin{array}{c} {\dot{z}}_{e_{1}}=z_{e_{2}}\\ {\dot{z}}_{e_{2}}=J\left(z\right)u+W\left(z\right)-\Delta \left(z,\delta ,\tau_{d}\right)-{\ddot{z}}_{d} \end{array}\right., \end{array}$$

For improvements in the overall control performance, our article develops an APPTMC with global finite-time stability for robots that ensures transient performance and Prescribed Control Performance (PCP) within the prescribed domain.

A subsection below discusses mathematical statements, assumptions, lemmas, and definitions that will confirm the stability and convergence of the APPTMC.

### 2.2. Related Definitions and Lemmas

Some notations are described as follows: ${\left[z\right]}^{0}=\mathrm{sign}\left(z\right)=\left\{\begin{array}{c} 1\;\;\;\mathrm{if}\;\;z>0\\ 0\;\;\mathrm{if}\;\;z=0\\ -1\;\;\mathrm{otherwise} \end{array}\right.$ and ${\left[z\right]}^{\phi }={\left|z\right|}^{\phi }\mathrm{sign}\left(z\right)$ with ϕ>0.

**Assumption** **1.** *Suppose that the desired trajectory $z_{d}$ and their higher order time derivatives are continuous and bounded.*

**Assumption** **2.** *Suppose that $\left|{\dot{\Delta }}_{i}\left(z,\delta ,\tau_{d}\right)\right|\leq {\bar{\Delta }}_{i}$, in which ${\bar{\Delta }}_{i}>0$ is a predefined positive constant, i=1,⋯,n.*

Consider the differential formula:

(4) $$\begin{array}{c} \dot{z}=f\left(z\left(t\right)\right),f\left(0\right)=0,z\left(0\right)=z_{0},z\in D \end{array}$$

where *f*: $D\to R^{n}$ is continuous.

**Definition** **1** ([47]). *It is defined that Equation (4)’s origin point is global finite time stable if the following two conditions are met: (1) Equation (4) is globally asymptotically stable; (2) any solution $z(z_{0},t)$ approach to the origin point at some finite time moments, i.e., $z(z_{0},t)=0$, $\forall t\geq T(z_{0})$, where $T(z_{0})$ presents the settling-time function.*

**Lemma** **1** ([37]). *Consider the following dynamic system:*

(5) $$\begin{array}{c} \left\{\begin{array}{c} {\dot{q}}_{0}=-\Pi_{1}\Psi_{q_{0}}+q_{1}\\ {\dot{q}}_{1}=-\Pi_{2}\Psi_{q_{1}}-\dot{\Delta } \end{array}\right. \end{array}$$

*where $\Psi_{q_{0}}$ and $\Psi_{q_{1}}$ are given by:*

$$\begin{array}{c} \left\{\begin{array}{c} \Psi_{q_{0}}={\left[q_{0}\right]}^{\frac{1}{2}}+A{\left[q_{0}\right]}^{\frac{3}{2}}\\ \Psi_{q_{1}}=\frac{1}{2}{\left[q_{0}\right]}^{0}+2Aq_{0}+\frac{3}{2}A^{2}{\left[q_{0}\right]}^{2} \end{array}\right. \end{array}$$

*If A>0, $\left|\dot{\Delta }\right|\leq \Delta_{\max}$, $\Delta_{\max}>0$ is a predefined positive constant, and $\Pi_{1}$ and $\Pi_{2}$ are selected in the set below:*

$$\begin{array}{c} \Pi =\left\{\left(\Pi_{1},\Pi_{2}\right)\in R^{2}\left|0<\Pi_{1}\leq 2\sqrt{\Delta_{\max}}\right.,\Pi_{2}>\frac{\Pi_{1}^{2}}{4}+\frac{4\Delta_{\max}^{2}}{\Pi_{1}^{2}}\right\}\cup \left\{\left(\Pi_{1},\Pi_{2}\right)\in R^{2}\left|\Pi_{1}>2\sqrt{\Delta_{\max}}\right.,\Pi_{2}>2\Delta_{\max}\right\}. \end{array}$$

Then $q_{0}=0$ and $q_{1}=0$ can be achieved in fixed time $T_{0}$ [37].

**Lemma** **2** ([48]). *Consider the differential formula with the following origin:*

(6) $$\begin{array}{c} {\left[Q^{\left(j\right)}\right]}^{\frac{\beta }{h-j}}+\lambda_{j-1}\left\{{\left[Q^{\left(j-1\right)}\right]}^{\frac{\beta }{h-j+1}}\left.+\ldots +\lambda_{2}\left[{\left[\ddot{Q}\right]}^{\frac{\beta }{h-2}}+\lambda_{1}\left({\left[\dot{Q}\right]}^{\frac{\beta }{h-1}}+\lambda_{0}{\left[Q\right]}^{\frac{\beta }{h}}\right)\right]\ldots \right\}=0\right., \end{array}$$

*If β is a positive scalar, h≥2 is an integer, and $\lambda_{k}$, (k=0,…,h−1) are chosen sufficiently large then, Equation (6) is finite-time stable for each j=1,…,h−1.*

**Lemma** **3** ([49]). *Consider the system:*

(7) $$\begin{array}{c} \left\{\begin{array}{c} \dot{\varpi }=-\nu_{1}\left(t\right){\left[\varpi \right]}^{1/2}-\nu_{2}\left(t\right)\varpi +\gamma \\ \dot{\gamma }=-\nu_{3}\left(t\right){\left[\varpi \right]}^{0}-\nu_{4}\left(t\right)\varpi +\chi \left(t\right) \end{array}\right.. \end{array}$$

*Suppose that $\left|\chi (t)\right|\leq \delta_{\chi }$ with unknown scalar $\delta_{\chi }\geq 0$. The time-varying gains $\nu_{m}\left(t\right)$, (m=1,2,3,4) are obtained by:*

(8) $$\begin{array}{c} \begin{array}{c} \nu_{1}\left(t\right)=\nu_{10}\sqrt{\rho_{0}\left(t\right)};\nu_{3}\left(t\right)=\nu_{30}\rho_{0}\left(t\right);\\ \nu_{2}\left(t\right)=\nu_{20}\rho_{0}\left(t\right);\nu_{4}\left(t\right)=\nu_{40}\rho_{0}^{2}\left(t\right), \end{array} \end{array}$$

*where positive constants $\nu_{m0}$ that satisfy the condition: $4\nu_{30}\nu_{40}\geq \left(8\nu_{30}+9\nu_{10}^{2}\right)\nu_{20}^{2}$. $\rho_{0}\left(t\right)$ is a positive function and is tuned by the below adaptive law:*

(9) $$\begin{array}{c} {\dot{\rho }}_{0}\left(t\right)=\left\{\begin{array}{c} \varepsilon \;\;\;\mathrm{if}\;\;\;\left|\varpi \right|\geq \delta_{\varpi }\\ 0\;\;\;\mathrm{otherwise} \end{array}\right., \end{array}$$

*where $\varepsilon ,\delta_{\varpi }$ is arbitrary positive scalar.*

*Thus, the states in Equation (7) converge towards the origin within a finite amount of time.*

## 3. Development of the Proposed Strategy

### 3.1. Design of an USOSMO

This subsection designs a USOSMO to estimate directly all uncertain terms. For bounded uncertain terms, the developed observer converges exactly in finite time, and also with a convergence time that is uniformly bounded for all initial conditions.

Using Equation (2), the observer is designed as follows:

(10) $$\begin{array}{c} \left\{\begin{array}{c} {\tilde{z}}_{2}=z_{2}-{\hat{z}}_{2}\\ {\dot{\hat{z}}}_{2}=J\left(z\right)u+W\left(z\right)-\hat{\Delta }+\theta_{1}\Psi_{1}\left({\tilde{z}}_{2}\right)\\ \dot{\hat{\Delta }}=-\theta_{2}\Psi_{2}\left({\tilde{z}}_{2}\right) \end{array}\right. \end{array}$$

where $\Psi_{1}\left({\tilde{z}}_{2}\right)$ and $\Psi_{2}\left({\tilde{z}}_{2}\right)$ are selected as:

(11) $$\begin{array}{c} \left\{\begin{array}{c} \Psi_{1}\left({\tilde{z}}_{2}\right)={\left[{\tilde{z}}_{2}\right]}^{\frac{1}{2}}+\alpha {\left[{\tilde{z}}_{2}\right]}^{\frac{3}{2}}\\ \Psi_{2}\left({\tilde{z}}_{2}\right)=\frac{1}{2}{\left[{\tilde{z}}_{2}\right]}^{0}+2\alpha {\tilde{z}}_{2}+\frac{3}{2}\alpha^{2}{\left[{\tilde{z}}_{2}\right]}^{2} \end{array}\right. \end{array}$$

$z_{2}$ has an approximate value of ${\hat{z}}_{2}$. $\theta_{1}$, $\theta_{2}$, and α represent user-designed parameters of observer. $\theta_{1}$ and $\theta_{2}$ are selected respectively with $\Pi_{1}$ and $\Pi_{2}$ in the set as stated in Lemma 1.

The following theorem describes the design procedure of the observer.

**Theorem** **1.** *The proposed observer’s estimate errors will converge towards zero in a fixed time regardless of the initial conditions and of bounded uncertain terms $\Delta \left(z,\delta ,\tau_{d}\right)$.*

**Proof of Theorem 1.**  The proposed observer’s estimate errors can be rewritten in the below expression.

(12) $$\begin{array}{c} \left\{\begin{array}{c} {\tilde{z}}_{2}=z_{2}-{\hat{z}}_{2}\\ \tilde{\Delta }=\hat{\Delta }-\Delta \end{array}\right. \end{array}$$

Taking time derivative of Equation (12) and using Equation (10) yields

(13) $$\begin{array}{c} \left\{\begin{array}{c} {\dot{\tilde{z}}}_{2}=-\theta_{1}\Psi_{1}\left({\tilde{z}}_{2}\right)+\tilde{\Delta }\\ \dot{\tilde{\Delta }}=-\theta_{2}\Psi_{2}\left({\tilde{z}}_{2}\right)-\dot{\Delta } \end{array}\right. \end{array}$$

where $\tilde{\Delta }$ represents the estimation error of the lumped uncertainty.

According to Lemma 1, it is concluded that the differentiator (13) is uniformly exact convergent, ${\tilde{z}}_{2}=0$ and $\tilde{\Delta }=0$ are achieved in fixed time $T_{0}$ regardless of the initial conditions and of bounded uncertain terms. For the sake of brevity, the definition of $T_{0}$ could be found in the study [37]. $T_{0}$ was defined in Equation (12), as an upper bound for the convergence time of any trajectory of Equation (3) in the study [37].

This proof is completed. □

**Remark** **1.** *Comparing with some recently proposed observers such as [16,36,39] we found that all three observers achieve only finite time convergence i.e., the convergence time of the observer depends on the initial condition whereas the proposed observer achieves uniform convergence in fixed time. In addition, refs. [16,36] require a measured value of the acceleration, which is not usually available, ref. [39] is known as a TOSMO and the feature of this observer is slow convergence. Therefore, the proposed observer can improve some shortcomings of the three observers.*

### 3.2. Design of the PPC

Based on the theory of the PPC, the tracking error $z_{e}$ is constrained to the following domain:

(14) $$\begin{array}{c} -P_{l}\left(t\right)<z_{e}\mathrm{sign}\left(z_{e}\left(0\right)\right)<P_{u}\left(t\right) \end{array}$$

where $z_{e}\left(0\right)$ is the initial error, the PPFs are $P_{u}\left(t\right)=\left(P_{0}-P_{\infty }\right)e^{-rt}+P_{\infty }$ and $P_{l}\left(t\right)=\left(P_{1}-P_{\infty }\right)e^{-rt}+P_{\infty }$, and the $P_{u}\left(t\right)$ and $P_{l}\left(t\right)$ are defined as: $P_{u}\left(t\right)$ and $P_{l}\left(t\right):R_{+}\to R_{+}$ are smoothly, positive, and decreasing functions which respectively satisfying $\lim_{t\to \infty }P_{u}\left(t\right)=P_{\infty }>0$, $\lim_{t\to \infty }P_{l}\left(t\right)=P_{\infty }>0$. $P_{0}>\left|z_{e}\left(0\right)\right|>0,P_{0}⩾P_{1}⩾P_{\infty }$, *r* are design constants to adjust the specified performance domain.

**Figure 1:**
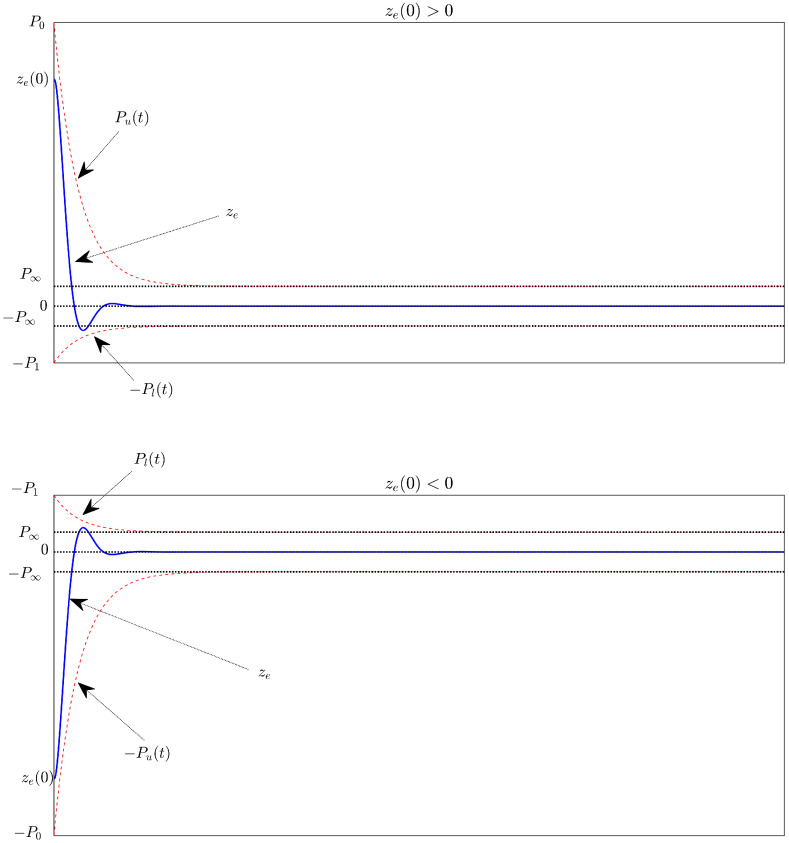
Description of the prescribed performance definition.

Different from the existing PPC studies [41,42,43,44,45,46], two separate PPFs including $P_{u}\left(t\right)$ and $P_{l}\left(t\right)$ are proposed to manage the tracking errors in our paper. When the sign of the initial error changes, the lower and upper bounds will be reversed through the signum function. $P_{u}\left(t\right)$ and $P_{l}\left(t\right)$ represent upper and lower bounds for the performance domain, respectively. The upper boundary $P_{u}\left(t\right)$ sets the maximum allowable tracking error $z_{e}$ at steady-state and limits the convergence rate while the lower boundary $P_{l}\left(t\right)$ sets the allowable maximum boundary of the overshoot and limits the allowable maximum size of the SSE $z_{e}$ at the lower boundary. Because both PPFs are set the same boundary of the control error at a steady state lead to the specified performance space is increased compared to the classical PPC. Furthermore, the SSE boundaries will be symmetrical to zero, so when the transformed error is zero, the tracking error will be as well. Using the above proposal, ETFs can be designed more easily. The designed ETF does not suffer from singularity issues. Figure 1 shows the description of the prescribed performance definition that is proposed in our paper.

**Remark** **2.** *It is prescribed that the allowable maximum size of tracking steady state error $z_{e}$ is $P_{\infty }$, that its maximum overshoot must be smaller than $P_{1}$, and that convergence rate of $z_{e}$ depends on the decreasing rate of $P_{u}\left(t\right)$ adjusted by r. The output trajectory of the system is determined by the appropriate selection of $P_{u}\left(t\right)$ and $P_{l}\left(t\right)$.*

The constrained error dynamics are converted to their equivalent unconstrained dynamics by the following ETF:

(15) $$\begin{array}{c} z_{e_{1}}=P\left(t\right)T\left(\varrho_{1}\right) \end{array}$$

where $\varrho_{1}$ is a transformed error, $T(\varrho_{1})$ is an ETF, and

$$\begin{array}{c} P\left(t\right)=\left\{\begin{array}{c} P_{u}\left(t\right)\;\;\;\mathrm{if}\;\mathrm{sign}\left(z_{e}.z_{e}\left(0\right)\right)>0\\ P_{l}\left(t\right)\;\;\;\mathrm{if}\;\mathrm{sign}\left(z_{e}.z_{e}\left(0\right)\right)<0 \end{array}\right.. \end{array}$$

$T(\varrho_{1})$ has the properties:

- it is a smooth and strictly increasing function;
$-1<T(\varrho_{1})<1$;
- $T(\varrho_{1})=0$ if $\varrho_{1}=0$;
- $\left\{\begin{array}{c} \lim_{\varrho_{1}\to -\infty }T\left(\varrho_{1}\right)=-1\\ \lim_{\varrho_{1}\to +\infty }T\left(\varrho_{1}\right)=1 \end{array}\right.$.

Considering all possible scenarios, as follows:

If $z_{e}\left(0\right)>0$ and $z_{e}>0$ then $0⩽T(\varrho_{1})<1$ and $P_{u}\left(t\right)>0$. Hence, $0⩽P_{u}\left(t\right)T\left(\varrho_{1}\right)<P_{u}\left(t\right)$; If $z_{e}\left(0\right)>0$ and $z_{e}<0$ then $-1<T(\varrho_{1})⩽0$ and $P_{l}\left(t\right)>0$. Hence, $-P_{l}\left(t\right)<P_{l}\left(t\right)T\left(\varrho_{1}\right)⩽0$. It is concluded that whenever $z_{e}\left(0\right)>0$, then $-P_{l}\left(t\right)<z_{e}<P_{u}\left(t\right)$.

If $z_{e}\left(0\right)<0$ and $z_{e}<0$ then $-P_{u}\left(t\right)<P_{u}\left(t\right)T\left(\varrho_{1}\right)<0$. If $z_{e}\left(0\right)<0$ and $z_{e}>0$ then $0<P_{l}\left(t\right)T\left(\varrho_{1}\right)<P_{l}\left(t\right)$. It is concluded that whenever $z_{e}\left(0\right)<0$ then $-P_{u}\left(t\right)<z_{e}<P_{l}\left(t\right)$

Consequently, Equation (14) can be obtained fully which means the tracking error behavior will be prescribed over transient and steady-state scenarios.

The ETF in Equation (15) is proposed as

(16) $$\begin{array}{c} T\left(\varrho_{1}\right)=\frac{2}{\pi }\arctan\left(\varrho_{1}\right) \end{array}$$

As a result, the transformed error $\varrho_{1}$ is given by:

(17) $$\begin{array}{c} \varrho_{1}=\tan\left(\frac{\pi z_{e_{1}}}{2P(t)}\right) \end{array}$$

Calculating the first-order derivative of $\arctan(\varrho_{1})$ with respect to time obtains

(18) $$\begin{array}{c} \begin{array}{c} {\left(\arctan(\varrho_{1})\right)}^{\prime }=\frac{{\dot{\varrho }}_{1}}{1+\varrho_{1}^{2}} \end{array} \end{array}$$

Using Equations (16) and (18), the first-order derivative of $z_{e_{1}}$ is

(19) $$\begin{array}{c} \begin{array}{c} {\dot{z}}_{e_{1}}=\dot{P}\left(t\right)T\left(\varrho_{1}\right)+P\left(t\right)\dot{T}\left(\varrho_{1}\right)\\ \;\;\;=\dot{P}\left(t\right)\frac{2}{\pi }\arctan\left(\varrho_{1}\right)+P\left(t\right)\frac{2}{\pi }\frac{{\dot{\varrho }}_{1}}{1+\varrho_{1}^{2}} \end{array} \end{array}$$

where $\dot{P}\left(t\right)=\left\{\begin{array}{c} {\dot{P}}_{u}\left(t\right)\;\;\mathrm{if}\;\;\;\mathrm{sign}\left(z_{e}.z_{e}\left(0\right)\right)>0\\ {\dot{P}}_{l}\left(t\right)\;\;\;\mathrm{if}\;\;\;\mathrm{sign}\left(z_{e}.z_{e}\left(0\right)\right)<0 \end{array}\right.$.

Therefore, the first-order derivative of $\varrho_{1}$ is derived from Equation (19):

(20) $$\begin{array}{c} {\dot{\varrho }}_{1}=\frac{\pi \left(1+\varrho_{1}^{2}\right)}{2P(t)}\left({\dot{z}}_{e_{1}}-\frac{2\dot{P}\left(t\right)}{\pi }\arctan\left(\varrho_{1}\right)\right) \end{array}$$

Calculating the second-order derivative of $\arctan(\varrho_{1})$ with respect to time obtains

(21) $$\begin{array}{c} {\left(\arctan(\varrho_{1})\right)}^{\prime \prime }=\frac{{\ddot{\varrho }}_{1}\left(1+\varrho_{1}^{2}\right)-2\varrho_{1}{{\dot{\varrho }}_{1}}^{2}}{{\left(1+\varrho_{1}^{2}\right)}^{2}} \end{array}$$

Using Equations (16), (18), and (21), the second-order derivative of $z_{e_{1}}$ is

(22) $$\begin{array}{c} \begin{array}{c} {\ddot{z}}_{e_{1}}={\left(\dot{P}\left(t\right)T\left(\varrho_{1}\right)+P\left(t\right)\dot{T}\left(\varrho_{1}\right)\right)}^{\prime }\;\\ \;\;\;=\ddot{P}\left(t\right)T\left(\varrho_{1}\right)+2\dot{P}\left(t\right)\dot{T}\left(\varrho_{1}\right)+P\left(t\right)\ddot{T}\left(\varrho_{1}\right)\\ \;\;=\frac{2}{\pi }\left(\ddot{P}\left(t\right)\arctan\left(\varrho_{1}\right)+\frac{2\dot{P}\left(t\right){\dot{\varrho }}_{1}}{1+\varrho_{1}^{2}}-\frac{2P\left(t\right)\varrho_{1}{{\dot{\varrho }}_{1}}^{2}}{{\left(1+\varrho_{1}^{2}\right)}^{2}}\right)+\frac{2P(t)}{\pi }\frac{{\ddot{\varrho }}_{1}}{\left(1+\varrho_{1}^{2}\right)} \end{array} \end{array}$$

where $\ddot{P}\left(t\right)=\left\{\begin{array}{c} {\ddot{P}}_{u}\left(t\right)\;\;\mathrm{if}\;\;\;\mathrm{sign}\left(z_{e}.z_{e}\left(0\right)\right)>0\\ {\ddot{P}}_{l}\left(t\right)\;\;\;\mathrm{if}\;\;\;\mathrm{sign}\left(z_{e}.z_{e}\left(0\right)\right)<0 \end{array}\right.$.

Therefore, the second-order derivative of $\varrho_{1}$ is derived from Equation (22):

(23) $$\begin{array}{c} {\ddot{\varrho }}_{1}=\frac{\pi \left(1+\varrho_{1}^{2}\right)}{2P(t)}\left({\ddot{z}}_{e_{1}}-\frac{2}{\pi }\left(\ddot{P}\left(t\right)\arctan\left(\varrho_{1}\right)+\frac{2\dot{P}\left(t\right){\dot{\varrho }}_{1}}{1+\varrho_{1}^{2}}-\frac{2P\left(t\right)\varrho_{1}{{\dot{\varrho }}_{1}}^{2}}{{\left(1+\varrho_{1}^{2}\right)}^{2}}\right)\right) \end{array}$$

with $\frac{\pi \left(1+e^{2}\right)}{2P(t)}>0$.

Referring Equations (3) and (23), the robot dynamics can be presented in unconstrained dynamics:

(24) $$\begin{array}{c} \left\{\begin{array}{c} {\dot{\varrho }}_{1}=\varrho_{2}\\ {\dot{\varrho }}_{2}=\Theta \left(J\left(z\right)u+W\left(z\right)-\Delta \left(z,\delta ,\tau_{d}\right)-{\ddot{z}}_{d}-\bar{P}\right) \end{array}\right.. \end{array}$$

where $\Theta =\frac{\pi \left(1+e^{2}\right)}{2P(t)}>0$ and $\bar{P}=\frac{2}{\pi }\left(\ddot{P}\left(t\right)\arctan\left(\varrho_{1}\right)+\frac{2\dot{P}\left(t\right){\dot{\varrho }}_{1}}{1+\varrho_{1}^{2}}-\frac{2P\left(t\right)\varrho_{1}{{\dot{\varrho }}_{1}}^{2}}{{\left(1+\varrho_{1}^{2}\right)}^{2}}\right)$.

### 3.3. Design of NISMS

A modified NISMS is proposed to control the transformed errors to be skated on its surface in finite time, as follows:

(25) $$\begin{array}{c} s=\varrho_{2}-\varrho_{2}\left(0\right)+\int_{0}^{t}{\left[\sigma_{1}\left({\left[\varrho_{2}\right]}^{\frac{\beta }{h-1}}+\sigma_{0}{\left[\varrho_{1}\right]}^{{}^{\frac{\beta }{h}}}\right)\right]}^{\frac{h-2}{\beta }}d\iota , \end{array}$$

where ι is the variable according to time, $\sigma_{0}$ and $\sigma_{1}$ are design constants. Due to its integral form, the proposed NISMS does not have any singularity issues.

If s=0 and $\dot{s}=0$, then the proposed system is in sliding mode. Equation (25) provides the following results:

(26) $$\begin{array}{c} \dot{\varrho_{2}}=-{\left[\sigma_{1}\left({\left[\varrho_{2}\right]}^{\frac{\beta }{h-1}}+\sigma_{0}{\left[\varrho_{1}\right]}^{{}^{\frac{\beta }{h}}}\right)\right]}^{\frac{h-2}{\beta }}. \end{array}$$

Then, Equation (26) can be presented in the following form:

(27) $$\begin{array}{c} \left\{\begin{array}{c} {\dot{\varrho }}_{1}=\varrho_{2}\\ {\left[{\ddot{\varrho }}_{1}\right]}^{\frac{\beta }{h-2}}+\sigma_{1}\left({\left[\varrho_{2}\right]}^{\frac{\beta }{h-1}}+\sigma_{0}\varrho_{1}\right)=0 \end{array}\right.. \end{array}$$

With β=h=3 and j=2, Equation (27) can be obtained the results as Equation (6); According to Lemma 2, for any initial states $\varrho_{0}$, the states ϱ(t) of the system (27) will approach the origin within a finite period. Therefore, for any initial states $z_{e}\left(0\right)$, the tracking errors $z_{e}\left(t\right)$ will also converge to its origin within a finite period.

**Remark** **3.** *As a result of designing the NISMS (25), the second-order sliding mode for s variable, i.e., $s=\dot{s}=0$ leads to a third-order sliding mode of $\varrho_{1}\left(t\right)$ variable, i.e., $\varrho_{1}=\varrho_{2}=\dot{\varrho_{2}}=0,\left(r=3\right)$. Therefore, the proposed controller can achieve 3-sliding accuracy even when measurement noise or sampling effects are existing [50].*

### 3.4. Proposed Controller Design

This subsection presents the process of the strategy being synthesized and its stability proof.

Calculating the first-order derivative of *s* and noting the dynamics (24) yields:

(28) $$\begin{array}{c} \dot{s}=\Theta \left(J\left(z\right)u+W\left(z\right)-\Delta \left(z,\delta ,\tau_{d}\right)-{\ddot{z}}_{d}-\bar{P}\right)+{\left[\sigma_{1}\left({\left[\varrho_{2}\right]}^{\frac{\beta }{h-1}}+\sigma_{0}{\left[\varrho_{1}\right]}^{{}^{\frac{\beta }{h}}}\right)\right]}^{\frac{h-2}{\beta }} \end{array}$$

The proposed strategy is designed with the control torques as follows:

(29) $$\begin{array}{c} u=-J^{-1}\Theta^{-1}\left(z\right)\left(u_{0}+u_{ob}+u_{astw}\right), \end{array}$$

where the term $u_{0}$ is designed as:

$$\begin{array}{c} \begin{array}{c} u_{0}=\Theta \left(W\left(z\right)-{\ddot{z}}_{d}-\bar{P}\right)+{\left[\sigma_{1}\left({\left[\varrho_{2}\right]}^{\frac{\beta }{h-1}}+\sigma_{0}{\left[\varrho_{1}\right]}^{{}^{\frac{\beta }{h}}}\right)\right]}^{\frac{h-2}{\beta }} \end{array}, \end{array}$$

the term $u_{ob}$ is obtained from the observer’s output as

$$\begin{array}{c} \begin{array}{c} u_{ob}=-\Theta \hat{\Delta } \end{array}, \end{array}$$

and the reaching term $u_{astw}$ is designed according to Lemma 2, as follows:

$$\begin{array}{c} u_{astw}=\nu_{1}\left(t\right){\left[s\right]}^{\frac{1}{2}}+\nu_{2}\left(t\right)s+\int_{0}^{t}\left[\nu_{3}\left(t\right){\left[s\right]}^{0}+\nu_{4}\left(t\right)s\right]d\iota . \end{array}$$

**Figure 2:**
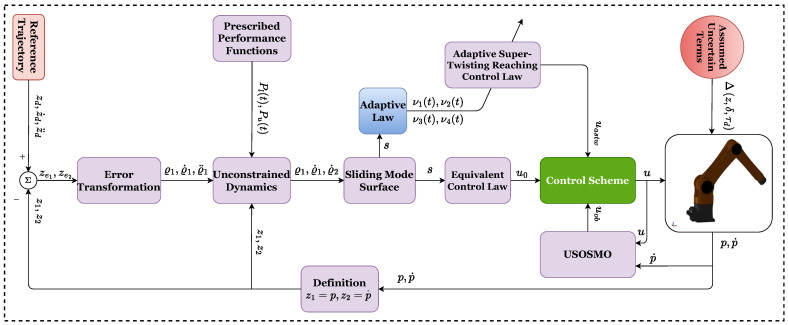
Algorithm diagram for the proposed control procedure.

Figure 2 illustrates the control system’s block diagram.

The below theorem summarizes the control design process.

**Theorem** **2.** *For the unconstrained system of the robot system, the sliding mode motions, s=0, $\varrho_{1}=0$, and $z_{e1}=0$, will take place in finite-time if the control torque (29) is designed based on the observer’s output (10), the proposed NISMS (25), and Lemma 3.*

**Proof of Theorem 2.**  Applying the control torque (29) to dynamic (28) obtains

(30) $$\begin{array}{c} \begin{array}{c} \dot{s}=\Theta \tilde{\Delta }-u_{astw}\\ \;\;\;\;\;=\Theta \tilde{\Delta }-\nu_{1}\left(t\right){\left[s\right]}^{\frac{1}{2}}-\nu_{2}\left(t\right)s-\int_{0}^{t}\left[\nu_{3}\left(t\right){\left[s\right]}^{0}+\nu_{4}\left(t\right)s\right]d\iota \end{array}. \end{array}$$

Dynamic (30) can be represented by:

(31) $$\begin{array}{c} \left\{\begin{array}{c} \dot{s}=-\nu_{1}\left(t\right){\left[s\right]}^{\frac{1}{2}}-\nu_{2}\left(t\right)s+\gamma \\ \dot{\gamma }=-\nu_{3}\left(t\right){\left[s\right]}^{0}-\nu_{4}\left(t\right)s+\dot{\Theta }\dot{\tilde{\Delta }} \end{array}\right.. \end{array}$$

where $\gamma =-\int_{0}^{t}\left[\nu_{3}\left(t\right){\left[s\right]}^{0}+\nu_{4}\left(t\right)s\right]d\iota +\Theta \tilde{\Delta }$. Suppose that $\left|\dot{\Theta }\dot{\tilde{\Delta }}\right|$ is bounded by $\left|\dot{\Theta }\dot{\tilde{\Delta }}\right|<K$ which is a Lipschitz continuous function according to time, K>0.

According to Lemma 3, the convergence of Equation (31) is finite time. Therefore, s=0 and γ=0 will be achieved within a finite amount of time. □

## 4. Simulations

The performance of the trajectory tracking motion control is simulated in this section to show the effectiveness of the APPTMC. Simulations were performed in MATLAB/SIMULINK environment to evaluate aspects including maximum overshoot, convergence index, transient response, and SSEs. In addition, approximation ability, chattering reduction, accuracy, and robustness of the control proposal also are considered thoroughly via comparison to other equivalent solutions including the SMC [7], the TSMC [29] and the FTSMC [29]. All controllers are applied to a 3-DOF robotic manipulator to investigate their effectiveness. The dynamic mathematics and kinematic design of this robot are derived from studies [2,51]. The system parameters of the robot are selected from [15,25]. In the studies [15,25], we describe in detail how the robot system was built using MATLAB/SIMULINK, and SOLIDWORKS software. In MATLAB/SIMULINK, the differential equations are solved using Euler’s method with a sampling time of $t_{s}=10^{-3}$.

### 4.1. Configuration of the Robot System and Control Parameter Selection

**Figure 3:**
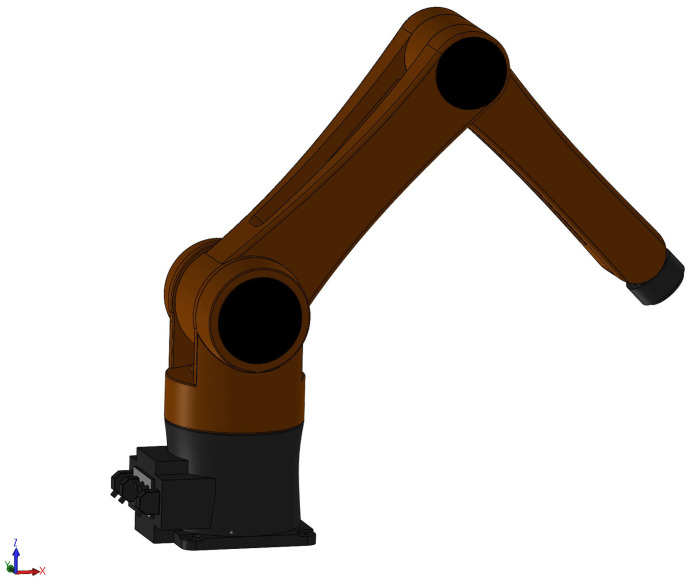
Geometric representation of the robot model.

The basic design parameters of the robot system including the length and weight of links, the center of mass, and inertia are reported in Table 2. A geometric representation of the robot model is shown in Figure 3.

Assigning a trajectory to the robot’s end-effector is the robot’s primary objective:

(32) $$\begin{array}{c} \left\{\begin{array}{c} X=0.85-0.01t\\ Y=0.2+0.2\sin(0.5t)\\ Z=0.7+0.2\cos(0.5t) \end{array}\right.\;\;\left(m\right). \end{array}$$

To evaluate the robustness and the effectiveness of the developed scheme in presence of uncertain terms including calculated-dynamical errors, frictions, and exterior disturbances, they are assumed in Table 3.

Following is a specific guide to choosing the control parameters.

**Remark** **4.** *The parameters of the proposed sliding surface including $\beta ,h,j,\sigma_{0},\sigma_{1}$ are chosen according to Lemma 2. The parameters of the term $u_{astw}$ including $\nu_{1},\nu_{2},\nu_{3}$ and $\nu_{4}$ are chosen according to Lemma 3. The parameters of the observer including $\theta_{1},\theta_{2}$ are chosen based on the set, as stated in Lemma 1 while α is chosen to be greater than zero. The parameters of the PPF including $P_{0},P_{1},P_{\infty },r$ are chosen to specify preset performance, as mentioned in Remark 1.*

Each controller’s parameters are selected to optimize performance within its capabilities. Accordingly, Table 4 provides the control parameters selected for each algorithm.

### 4.2. Simulation Results and Discussion

**Figure 4:**
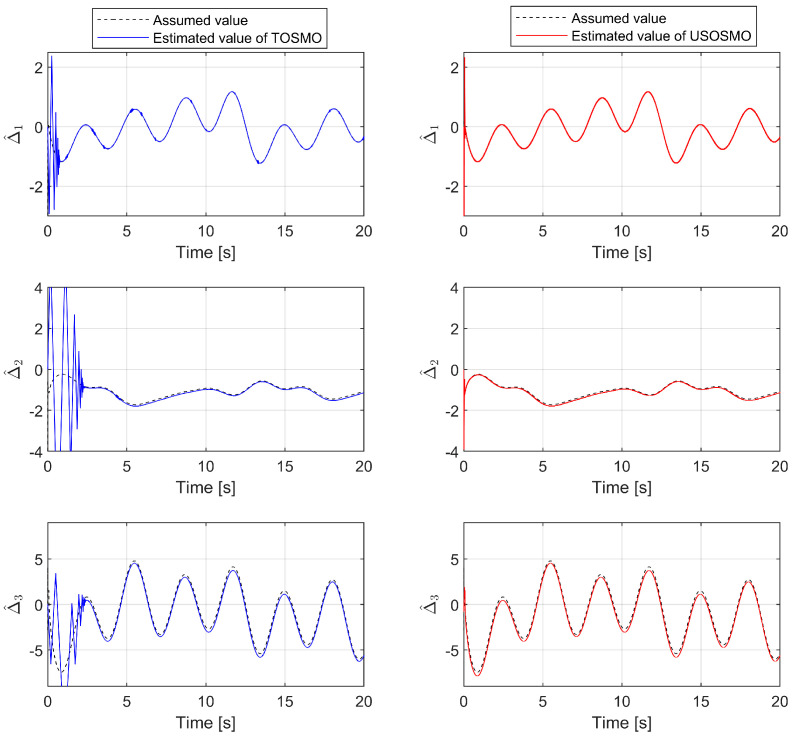
The description of performance estimation from the FnTDO and the proposed FxTDO.

**Figure 5:**
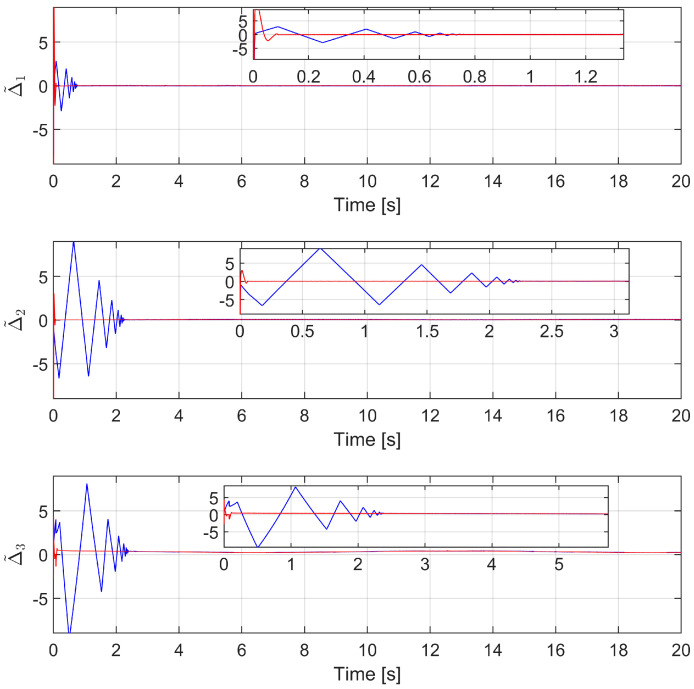
The comparison of the estimated errors between the FnTDO and the proposed FxTDO.

We first investigate the efficiency and approximation of the proposed observer. We compare the estimation accuracy of the proposed FxTDO (USOSMO) with that of the FnTDO (TOSMO) [39]. The description of performance estimation from the FnTDO and the proposed FxTDO can be found in Figure 4. The estimated errors of the two observers are also plotted in Figure 5 to facilitate comparisons between them. According to Figure 4 and Figure 5, both observers seem to achieve the same good accuracy. However, the proposed observer provides much faster convergence than the FnTDO. The convergence of the FnTDO was achieved in finite time, thus, the FnTDO depended on the initial value. In contrast, the proposed FxTDO provided fixed-time uniform convergence of the estimation errors. The displayed advantages of the proposed observer have a major contribution to improving overall control performance for robot manipulators.

**Figure 6:**
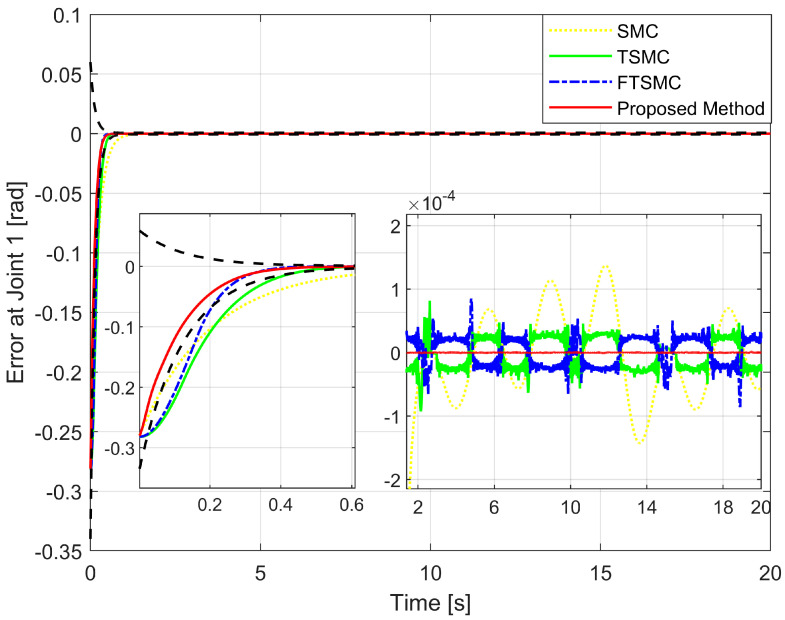
Tracking error of the first joint versus the desired trajectory.

**Figure 7:**
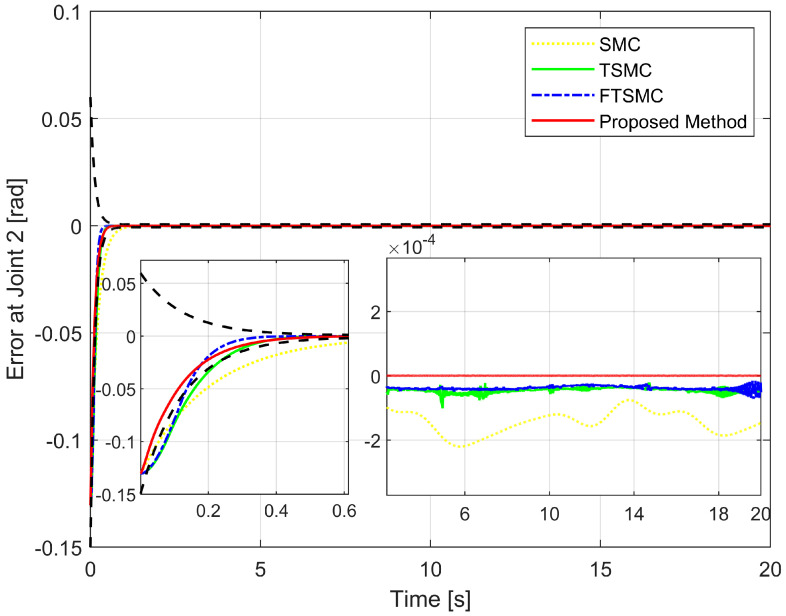
Tracking error of the second joint versus the desired trajectory.

**Figure 8:**
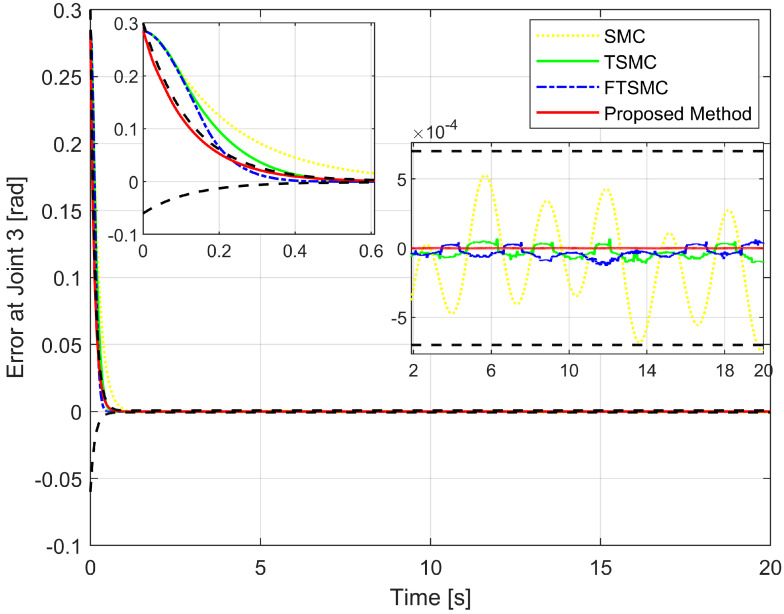
Tracking error of the third joint versus the desired trajectory.

We will then investigate the simulation results in terms of regulatory issues and tracking issues. Based on the results displayed in Figure 6, Figure 7 and Figure 8, we analyze the regulation problem.

For a fair investigation, the system states are considered with the same initial conditions. We investigate two terms in the approach stage (from the 0th second to the 0.6th second), including convergence rate and maximum overshoot, and find that the proposed strategy fulfills these both performance indices with a prescribed performance defined by Equation (14). By adjusting the design parameters including $P_{0},P_{1},P_{\infty }$, and *r* we can control the output trajectory of the system within a predefined performance domain as described in Remark 2. However, the zoomed-in portions of Figure 6, Figure 7 and Figure 8 clearly show that none of the other three methods satisfy both of the above performance indices.

Consider the trajectory tracking problems when controlling the robotic arm to follow the desired trajectory, as stated in Equation (32). Tracking accuracy and control performance can be evaluated by analyzing SSEs after the convergence period to equilibrium. Therefore, the time used to calculate the SSE can be calculated from the 2nd to 20th seconds through the Roots-Mean-Square Method (RMSM) as introduced below.

(33) $$\begin{array}{c} E_{X}=\sqrt{\frac{1}{S}\sum_{i=1}^{S}{\left|\left(X_{ri}-X_{i}\right)\right|}^{2}};E_{Y}=\sqrt{\frac{1}{S}\sum_{i=1}^{S}{\left|\left(Y_{ri}-Y_{i}\right)\right|}^{2}};E_{Z}=\sqrt{\frac{1}{S}\sum_{i=1}^{S}{\left|\left(Z_{ri}-Z_{i}\right)\right|}^{2}};\\ E_{1}=\sqrt{\frac{1}{S}\sum_{i=1}^{S}{\left|\left(p_{r1i}-p_{1i}\right)\right|}^{2}};E_{2}=\sqrt{\frac{1}{S}\sum_{i=1}^{S}{\left|\left(p_{r2i}-p_{2i}\right)\right|}^{2}};E_{3}=\sqrt{\frac{1}{S}\sum_{i=1}^{S}{\left|\left(p_{r3i}-p_{3i}\right)\right|}^{2}}, \end{array}$$

where S denotes the number of the calculated samples. Roots-Mean-Square Errors (RMSEs) for joint 1, joint 2, and joint 3 are $E_{1}$, $E_{2}$, and $E_{3}$, respectively. RMSEs for X axis,Y axis, and Z axis are $E_{X}$$E_{Y}$, and $E_{Z}$ respectively. ${\left[X_{i},Y_{i},Z_{i}\right]}^{T}$ denotes the actual position and ${\left[X_{ri},Y_{ri},Z_{ri}\right]}^{T}$ denotes the reference position at time index *i*. ${\left[p_{1i},p_{2i},p_{3i}\right]}^{T}$ denotes the actual joint angle and ${\left[p_{r1i},p_{r2i},p_{r3i}\right]}^{T}$ denotes the reference joint angle at time index *i*.

**Figure 9:**
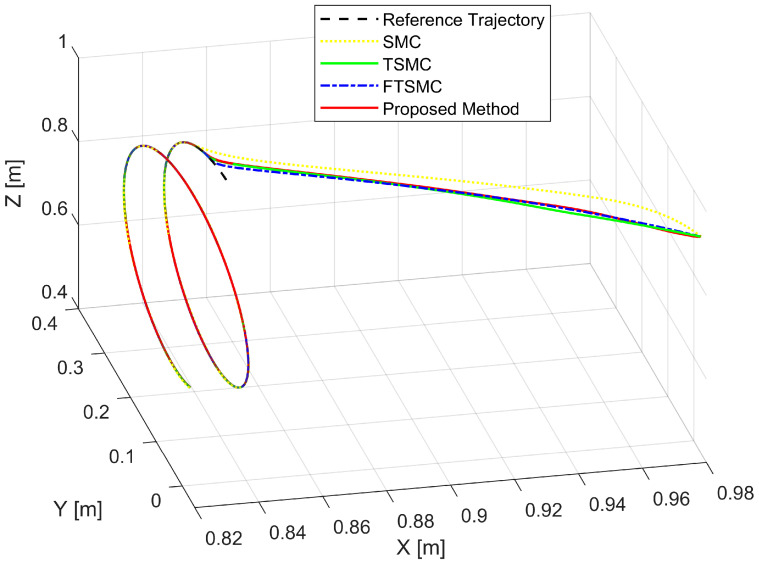
The real trajectories under all controllers versus the desired trajectory.

**Figure 10:**
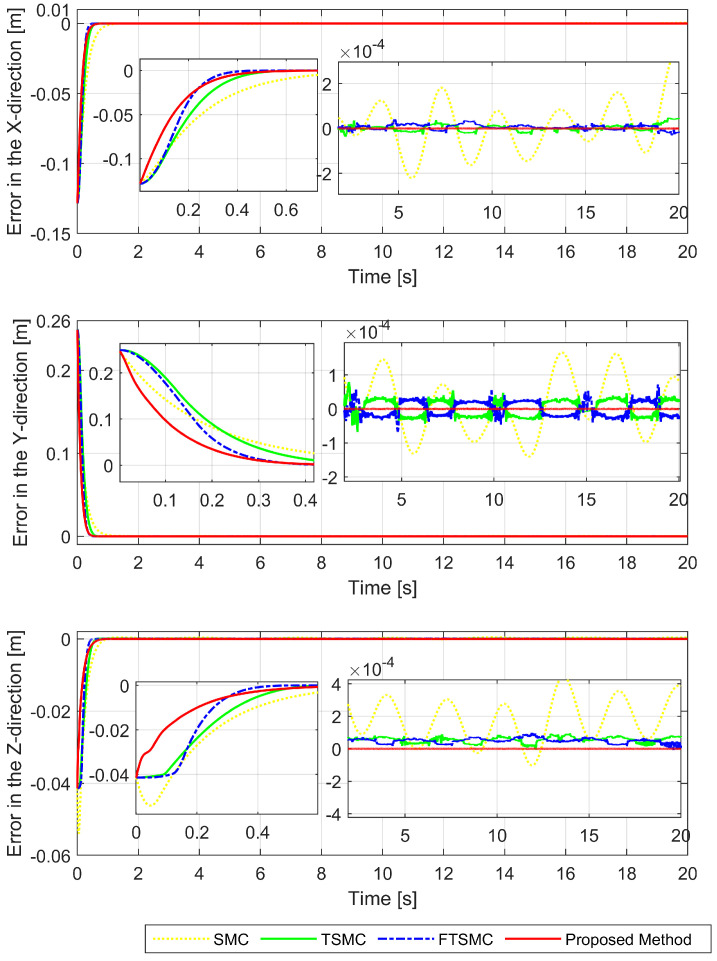
X-axis, Y-axis, and Z-axis error comparisons between the position of the end effector and the reference trajectory.

Figure 9 depicts the trajectory of the effective point of the robot arm separately controlled by four different methods. It is generally possible to control the robotic arm using each of the four methods to complete orbital tracking well. According to Figure 6, Figure 7 and Figure 8, tracking errors are compared between the real robot trajectory and the reference trajectory at each joint. Based on Figure 10, the end effector’s position and the reference trajectory are compared in terms of X-axis, Y-axis, and Z-axis errors. Using RMSE levels for joint errors, X-axis, Y-axis, and Z-axis errors, tracking accuracy was evaluated. The results pointed in Figure 6, Figure 7, Figure 8 and Figure 10, and Table 5 show that the proposed strategy has obtained the highest tracking accuracy and the smallest steady-state errors. Overall, both controllers including TSMC, and FTSMC have proven their effectiveness in trajectory tracking when they could provide relatively high tracking accuracy. Their SSEs can be within predetermined performance boundaries while the SSEs of the SMC sometimes cross performance boundaries.

**Figure 11:**
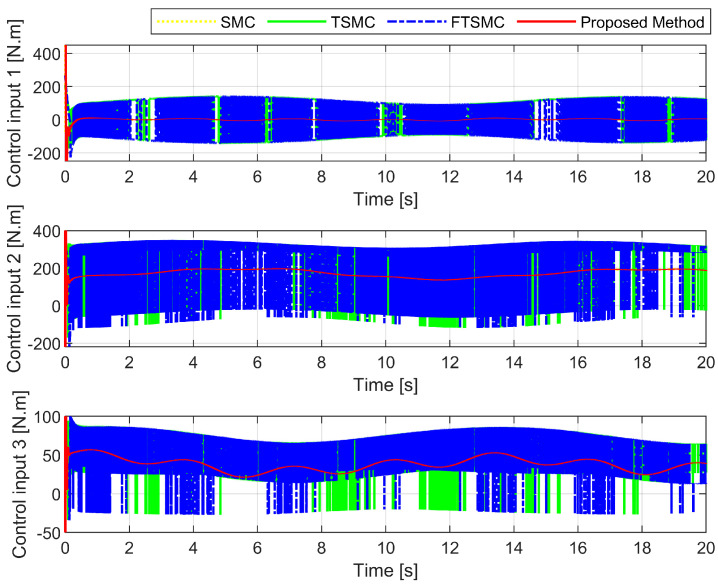
The control torque of the four different strategies.

Figure 11 shows the control torque provided by the four different control schemes. The proposed scheme achieved smoother control torques for the robot as a result of estimating uncertainty terms from observers and using the ASTwCL for the reaching phase, as well as robustness that allowed it to cope with the effects of uncertain elements and preserve tracking precision despite uncertain components. As a result of the application of a high-frequency reaching control law, the three remaining control schemes produced control torques with harmful chattering phenomena. Although those control schemes still guarantee robustness as well as provide a good level of tracking performance. In reality, chattering may result in arm vibrations, moving parts in actuators, mechanical abrasions, and even heat generation in the controlled systems [13,52]. Therefore, chattering should be removed/reduced its effects.

**Figure 12:**
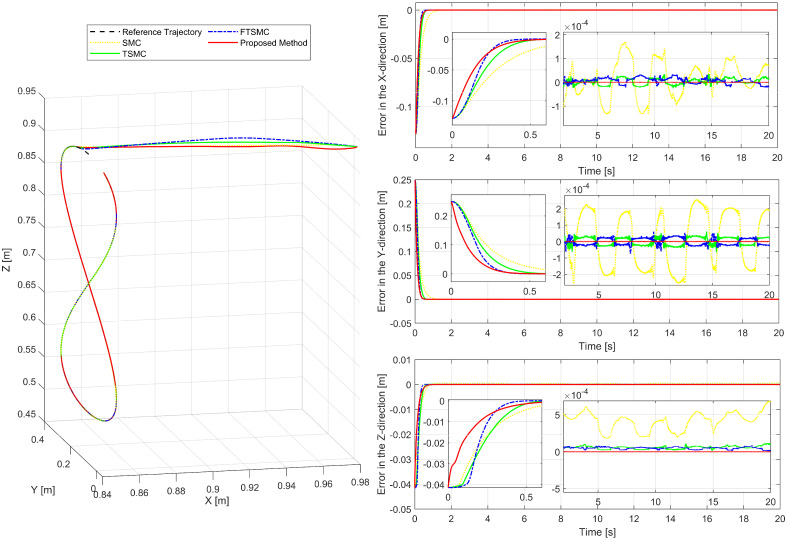
Performance of the control system in tracking another trajectory.

To prove the universality of the algorithm, the robot manipulator is controlled to follow a different trajectory. This trajectory tracking performance of the robot is presented in Figure 12. Through the obtained simulation results, we observed that they have the results as those of the first example. Therefore, to avoid repeated analysis, we only present briefly the tracking control performance as shown in Figure 12.

## 5. Conclusions

The proposed APPTMC with the capability of obtaining prescribed performance has been presented to solve the tracking control problem of robot manipulators under the influence of disturbances and dynamical uncertainties. The modified PPFs have been proposed to manipulate position tracking errors in a pre-designed performance domain. Especially, the SSE boundaries will be symmetrical to zero with the modified PPFs, so when the transformed error is zero, the tracking error will be as well. A new NISMS based on the transformed errors allows knowing the allowable maximum size of the control errors in the steady-state, finite-time convergence speed, and singularity elimination. A fixed-time USOSMO was proposed to directly estimate the lumped uncertainty. The integration of the designed USOSMO, the suggested sliding mode surface based on the transformed errors, and the transformed errors formed an APPTMC for robotic manipulators with global finite-time stability. The developed control solution provided prescribed performance, chattering reduction ability, and robustness in coping with the effects of uncertain elements. The stability of the whole closed-loop system of the tracking control method has been carried out by Lyapunov theory. The effectiveness and robustness of the proposed method have been fully confirmed through numerical simulations.

We examined the robot system in our paper with matched uncertain terms, including dynamic uncertainties, external disturbances, and frictions. Therefore, we plan to extend the consideration of time-varying mismatched as well as time-varying matched uncertainties to robot systems in the future. 

## Figures and Tables

**Table 1:** List of nomenclature.

Description	Notation
the real *n*-dimensional space	$R^{n}$
the set of *m* by *n* real matrices	$R^{n\times m}$
the transpose of	$\cdot^{T}$
Euclidean norm of	∥·∥
absolute value of	|·|
vector of joint angular acceleration	$\ddot{p}\in R^{n\times 1}$
vector of joint angular velocity	$\dot{p}\in R^{n\times 1}$
vector of joint angular position	$p\in R^{n\times 1}$
vector of system state	$z={\left[z_{1},z_{2}\right]}^{T}={\left[p,\dot{p}\right]}^{T}\in R^{n\times 1}$
vector of tracking error	$z_{e}={\left[z_{e_{1}}^{T},z_{e_{2}}^{T}\right]}^{T}\in R^{2n\times 1}$
vector of the desired trajectory	$z_{d}\in R^{n\times 1}$
vector of NISMS	$s\in R^{n\times 1}$
the first-order derivative of *x*	$\dot{x}$
the second-order derivative of *x*	$\ddot{x}$
Euler’s number	e

**Table 2:** Basic design parameters of a 3-DOF robot system.

Description	Link 1	Link 2	Link 3
Link Length (m)	$l_{1}=0.25$	$l_{2}=0.7$	$l_{3}=0.6$
Link Weight (kg)	$m_{1}=33.429$	$m_{2}=34.129$	$m_{3}=15.612$
Center of Mass (mm)	$\begin{array}{c} l_{c1x}=0\\ l_{c1y}=0\\ l_{c1z}=-0.7461 \end{array}$	$\begin{array}{c} l_{c2x}=0.3477\\ l_{c2y}=0\\ l_{c2z}=0 \end{array}$	$\begin{array}{c} l_{c3x}=0.3142\\ l_{c3y}=0\\ l_{c3z}=0 \end{array}$
Inertia (kg.m${}^{2}$)	$\begin{array}{c} I_{1xx}=0.7486\\ I_{1yy}=0.5518\\ I_{1zz}=0.5570 \end{array}$	$\begin{array}{c} I_{2xx}=0.3080\\ I_{2yy}=2.4655\\ I_{2zz}=2.3938 \end{array}$	$\begin{array}{c} I_{3xx}=0.0446\\ I_{3yy}=0.7092\\ I_{3zz}=0.7207 \end{array}$

**Table 3:** Assumed Uncertain Terms.

Type of the Assumed Uncertainty	Functions
Calculated-Dynamical Errors	$\delta H\left(p\right)=0.2H\left(p\right)$
	$\delta C\left(p,\dot{p}\right)=0.2C\left(p,\dot{p}\right)$
	$\delta g\left(p\right)=0.2g\left(p\right)$
Frictions $F_{r}\left(\dot{p}\right)\;\left(N.m\right)$	$F_{r1}\left(\dot{p}\right)=0.1\mathrm{sign}\left({\dot{p}}_{1}\right)+2{\dot{p}}_{1}$
	$F_{r2}\left(\dot{p}\right)=0.1\mathrm{sign}\left({\dot{p}}_{2}\right)+2{\dot{p}}_{2}$
	$F_{r3}\left(\dot{p}\right)=0.1\mathrm{sign}\left({\dot{p}}_{3}\right)+2{\dot{p}}_{3}$
Exterior Disturbances $\tau_{d}\;\left(N.m\right)$	$\tau_{d1}=4\sin(t)$
	$\tau_{d2}=5\sin(t)$
	$\tau_{d3}=6\sin(t)$

**Table 4:** Control parameter selection for the proposed scheme.

Description	Symbol	Value
USOSMO (10)	$\theta_{1},\theta_{2},\alpha$	$10,60,2\sqrt{30}$
PPF (14)	$P_{0},P_{1},P_{\infty },r$	0.023,0.006,0.0015,3
NISMS (25)	$\beta ,h,j,\sigma_{0},\sigma_{1}$	3,3,2,50,10
Proposed Control Law (29)	$\varepsilon ,\nu_{10},\nu_{20},\nu_{30},\nu_{40}$	3,2,6,10,100

**Table 5:** RMSEs via four Control Strategies.

Control System	$E_{X}$	$E_{Y}$	$E_{Z}$	$E_{1}$	$E_{2}$	$E_{3}$
SMC [7]	$\begin{array}{c} 1.1565\times 10^{-4} \end{array}$	$\begin{array}{c} 8.4785\times 10^{-5} \end{array}$	$\begin{array}{c} 2.1955\times 10^{-4} \end{array}$	$\begin{array}{c} 6.6134\times 10^{-5} \end{array}$	$\begin{array}{c} 1.4889\times 10^{-4} \end{array}$	$\begin{array}{c} 3.3847\times 10^{-4} \end{array}$
TSMC [29]	$\begin{array}{c} 1.4363\times 10^{-5} \end{array}$	$\begin{array}{c} 2.4533\times 10^{-5} \end{array}$	$\begin{array}{c} 5.8271\times 10^{-5} \end{array}$	$\begin{array}{c} 2.5713\times 10^{-5} \end{array}$	$\begin{array}{c} 4.6512\times 10^{-5} \end{array}$	$\begin{array}{c} 5.1967\times 10^{-5} \end{array}$
FTSMC [29]	$\begin{array}{c} 1.3054\times 10^{-5} \end{array}$	$\begin{array}{c} 2.2247\times 10^{-5} \end{array}$	$\begin{array}{c} 5.2373\times 10^{-5} \end{array}$	$\begin{array}{c} 2.3968\times 10^{-5} \end{array}$	$\begin{array}{c} 3.9330\times 10^{-5} \end{array}$	$\begin{array}{c} 5.0069\times 10^{-5} \end{array}$
Proposed Controller	$\begin{array}{c} 1.2158\times 10^{-7} \end{array}$	$\begin{array}{c} 2.9631\times 10^{-7} \end{array}$	$\begin{array}{c} 2.2370\times 10^{-7} \end{array}$	$\begin{array}{c} 3.4814\times 10^{-7} \end{array}$	$\begin{array}{c} 2.3686\times 10^{-7} \end{array}$	$\begin{array}{c} 1.9566\times 10^{-7} \end{array}$

## Data Availability

The data sets generated and/or analyzed during the current study are available from the corresponding author on reasonable request.

## Abbreviations

The following abbreviations are used in this manuscript:

CTC	Computed Torque Control
ACM	Adaptive Control Method
BsCM	Back-stepping Control Method
SMC	Sliding Mode Control
ISMC	Integral Sliding Mode Control
SSE	Steady-State Error
SOSMC	Second-Order Sliding Mode Control
TSMC	Terminal Sliding Mode Control
NTSMC	Non-singular Terminal Sliding Mode Control
FTSMC	Fast Terminal Sliding Mode Control
FNTSMC	Fast Non-singular Terminal Sliding Mode Control
NISMS	Nonsingular Integral Sliding Mode Surfac
FnTCM	Finite-Time Control Method
FxTCM	Fixed-Time Control Method
DO	Disturbance Observer
FnTDO	Finite-Time Disturbance Observer
FxTDO	Fixed-Time Disturbance Observer
SOSMO	Second-Order Sliding Mode Observer
USOSMO	Uniform Second-Order Sliding Mode Observer
TOSMO	Third-Order Sliding Mode Observer
ASTwCL	Adaptive Super-twisting Control Law
PPC	Prescribed Performance Control
PCP	Prescribed Control Performance
PPF	Prescribed Performance Function
ETF	Error Transformation Function
DOF	Degrees of Freedom
RMSM	Roots-Mean-Square Method
RMSE	Roots-Mean-Square Error
SMO-CM	Sliding Mode Observer-based Control Method
TDE-CM	Time-Delay Estimation-based Control Method
DO-CM	Disturbance Observer-based Control Method
ADRCM	Active Disturbance Rejection Control Method
APPTMC	Adaptive Prescribed Performance Tracking Motion Control
